# New Technologies for the Understanding, Assessment, and Intervention of Emotion Regulation

**DOI:** 10.3389/fpsyg.2019.01261

**Published:** 2019-06-18

**Authors:** Desirée Colombo, Javier Fernández-Álvarez, Azucena García Palacios, Pietro Cipresso, Cristina Botella, Giuseppe Riva

**Affiliations:** ^1^Department of Basic Psychology, Clinical and Psychobiology, Jaume I University, Castellón de la Plana, Spain; ^2^Department of Psychology, Università Cattolica del Sacro Cuore, Milan, Italy; ^3^CIBER of Physiopathology of Obesity and Nutrition, Madrid, Spain; ^4^Applied Technology for Neuro-Psychology Laboratory, Istituto Auxologico Italiano (IRCCS), Milan, Italy

**Keywords:** emotion regulation, virtual reality, biofeedback, internet interventions, smartphones, serious games

## Abstract

In the last decades, emotion regulation (ER) received increasing attention and became one of the most studied topics within the psychological field. Nevertheless, this construct has not been fully updated with the latest technological advancements. In this perspective, we will show how diverse technologies, such as virtual reality (VR), wearable biosensors, smartphones, or biofeedback techniques, can be applied to the understanding, assessment, and intervention of ER. After providing a brief overview of the currently available technological developments, we will discuss the benefits of incorporating new technologies in ER field, including ecological validity, intervention personalization, and the integration of understudied facets of ER, such as the implicit and interpersonal dimension.

## Introduction

Mental health, understood as “*a state of well-being in which every individual realizes his or her own potential, can cope with the normal stresses of life, can work productively and fruitfully, and is able to make a contribution to her or his community*” (World Health Organization, 2004), is witnessing a revolution due to the incorporation of new digital technologies. Although its real impact is difficult to foresee, it is a matter of fact that profound changes are already taking place at multiple levels. In this direction, different technologies have already been applied to the mental health realm. Illustrative examples are internet-based interventions (Andersson, 2016), mobile health (Firth et al., 2017; Grist et al., 2017), or the emerging field of mixed realities (MR) (Baus and Bouchard, 2014), serious games (Fleming et al., 2017), and biofeedback techniques (Schoenberg and David, 2014). ER is not the exception to the rule, and new technologies are called to transform our understanding of the field, and thus to positively impact on its assessment and intervention.

In the last years, ER emerged as one of the most studied constructs in the psychological field (Fernández-Álvarez et al., 2018a). ER is a dynamic process that every person implements with the aim of downregulating or upregulating positive and negative emotions in order to reach desirable states (Gross, 1998, 2015a). ER is always addressed to accomplish a certain goal (Tamir, 2016), either hedonic (i.e., maximize pleasure and/or minimize pain in the short term) or instrumental (i.e., maximize pleasure and/or minimize pain in the long term), by means of strategies (Gross and Jazaieri, 2014) that can be implemented before or after emotions’ occurrence (Gross, 1998). ER can be deployed both intrapersonally or interpersonally (Zaki and Craig Williams, 2013): people may try to regulate emotions in solitude, for instance, by reappraising a situation, but they can also modulate emotions interpersonally, for example, by seeking support from an intimate partner. Furthermore, ER can be explicit or implicit, as well as controlled or automatic (Braunstein et al., 2017). On one hand, ER is explicit when significant goals are deliberately pursued, and it is implicit when regulatory mechanisms are automatically activated by unconscious goals. On the other hand, automatic ER is a nonconscious attempt to regulate emotions, whether controlled ER involves top-down control mechanisms.

ER entails cognitive, behavioral, and physiological processes. From a *cognitive* point of view, among the most studied ER strategies, there are rumination, cognitive reappraisal, suppression, acceptance, savoring, and dampening (Naragon-Gainey et al., 2017). The generation of emotions is also intimately related to *behaviors*, which are indeed driven by our emotional states: people tend to engage in mood-increasing activities when feeling upset and join useful rather than pleasant activities when in a good mood (Taquet et al., 2016). Finally, a long tradition of research focused on the *psychophysiological* dimension of ER processes, both at a peripheral and neural level. The bed nucleus, the habenula, the striatum and the amygdala are the central cortical areas, while the prefrontal cortex (PFC), in particular, dorsolateral PFC, ventrolateral PFC, and ventromedial regions (vmPFC), and the anterior cingulate cortex constitute key subcortical regions (Ochsner et al., 2012; Lopez et al., 2018). Meanwhile, the autonomous system has also been extensively studied. A central process within this research line is the ECG activity, through which heart rate variability (HRV) can be calculated. In particular, the high frequency domain, related to the respiratory sinus arrhythmia, is considered to be an index of the vagal activity which in turn is a key for stress patterns and emotion regulation processes (Balzarotti et al., 2017).

Traditionally, ER has been measured by means of self-report questionnaires that consider ER as a stable trait of a person. Clear examples are the Emotion Regulation Questionnaire (ERQ), a 10-items self-report to assess the use of cognitive reappraisal and expressive suppression to regulate emotions (Gross and John, 2003), or the Difficulties in Emotion Regulation Questionnaire (DERS), which explores six different ER dimensions of emotion dysregulation (Gratz and Roemer, 2004). More recently, state questionnaires have been developed that can be applied to explore the adoption of single (Ganor et al., 2018; Marchetti et al., 2018) or multiple strategies (Katz et al., 2017; Lavender et al., 2017) in specific situations.

In the last years, emotion dysregulation has been shown to be a transdiagnostic factor (Kring and Sloan, 2009; Aldao et al., 2016; Fernandez et al., 2016; Fernández-Álvarez et al., 2018a,b). Ample evidence has suggested that poor regulatory skills constitute a vulnerability and maintenance feature among a wide range of mental disorders (Rottenberg et al., 2005; Mennin and Farach, 2007; Nolen-Hoeksema et al., 2008). The constant deployment of nonadaptive strategies to regulate emotions would elicit negative psychological health outcomes (Aldao et al., 2010; Sheppes et al., 2015; Urzúa et al., 2016). This lack of adaptiveness should not be understood as the utilization of specific strategies, but rather as an inflexible pattern in which the deployed strategies are incorrectly selected, inaccurately deployed, or unsuccessfully monitored (Gross, 2015b). Accordingly, the action control perspective states that emotion dysregulation is the result of failures in identifying when to regulate, how to do it, and how to deploy the selected strategy (Webb et al., 2012). Beyond psychopathology, the ability to adopt a wide repertoire of strategies with high variability across different situations (i.e., ER flexibility) (Aldao et al., 2015) has been shown to play a key role for mental well-being (Aldao and Nolen-Hoeksema, 2012; Bonanno and Burton, 2013).

## Assessing and Capturing Emotion Regulation Through New Digital Technologies

The value of using new technologies for the understanding and assessment of ER relies undoubtedly on the possibility of increasing ecological validity and exploring this process in real-life, thus overcoming the barriers of traditional laboratory/clinical settings and leading to the exploration of new facets of this process. In the next paragraphs, we will briefly discuss the current state-of-the-art of smartphones, smartphone-embedded sensors, and wearable biosensors application within the field, showing the potentialities of these tools to assess ER and to understand its temporal dynamics in daily life as well as the role of contextual and momentary factors. Furthermore, we will suggest virtual reality (VR) as an innovative approach to understand and assess ER that gives researchers the opportunity to develop realistic scenarios in which to elicit emotions and explore the way, ER is deployed.

### Ecological Momentary Assessment

In the last decade, an increasing number of Ecological Momentary Assessment (EMA) (Csikszentmihalyi and Larson, 1987; Shiffman et al., 2008) has been developed for the investigation and understanding of ER. By means of portable devices like Personal Digital Assistants (PDAs) and smartphones, EMA gave researchers the opportunity to observe and repeatedly assess people in daily life by providing prompted self-reports directly on electronic devices.

Altogether, the use of EMA through mobile devices significantly increased the knowledge about ER, shedding new light on the complexity of this process and on aspects that were still understudied. ER is indeed a dynamic process affected by situational, contextual, and momentary factors (Aldao, 2013; Doré et al., 2016), and strategy implementation in daily life only moderately correlates with ER trait measures (Brockman et al., 2017). This suggests that ER cannot be completely grasped and understood in traditional laboratory experiments. In that sense, EMA has been proposed as an innovative approach to explore ER (Bylsma and Rottenberg, 2011) in order to capture the temporal deployment of strategies and their impact on subsequent mood (Heiy and Cheavens, 2014; Catterson et al., 2017; Richardson, 2017), as well as the role of momentary affect (Brockman et al., 2017; Li et al., 2017b) and environmental factors (Heiy and Cheavens, 2014; English et al., 2017) in implementing certain strategies.

EMA could constitute a powerful tool not only for the understanding, but also for the assessment of ER. So far, many questionnaires have been developed, which measure strategies deployment, considering ER as a stable trait of a person, and thus underestimating the role of contextual and momentary factors. EMA could instead substitute or integrate classical paper-and-pencil, retrospective questionnaires by assessing ER directly in daily life. In turn, this would help clinicians to identify strategies that are to be targeted in the therapeutic process, as well as recognizing triggers and/or consequences of maladaptive strategies implementation on patients’ life (see, for example Anestis et al., 2010; Czyz et al., 2018). Nevertheless, the available literature on EMA and ER is limited to the research field, and we are not aware of studies applying EMA to assess ER in real clinical practice.

There are still many challenges ahead for EMAs. First of all, most of the studies assessing ER with EMA only rely on self-reports, assuming people to be perfectly able to recognize how they regulate emotions. Furthermore, standardized and *ad hoc* items to be implemented in mobile devices for the assessment of ER are currently not available, making it difficult to compare results across studies or to apply this approach in clinical practice, as well as specific guidelines for EMA designs to increase users’ adherence and reduce dropout rates (Colombo et al., 2018). Even if still not validated, a set of 20 self-regulation items specific for EMA are currently being developed (Eisenberg et al., 2017). Starting from 594 self-regulation survey items, Eisenberg and colleagues used the item response theory to choose a smaller set of items to specifically assess momentary self-regulation processes through EMA. To the best of our knowledge, this is the first attempt in this direction, leading the way to the possibility of developing standardized, specific items for the assessment of ER by means of EMA.

### Sensors and Wearable Biosensors

**Figure 1:**
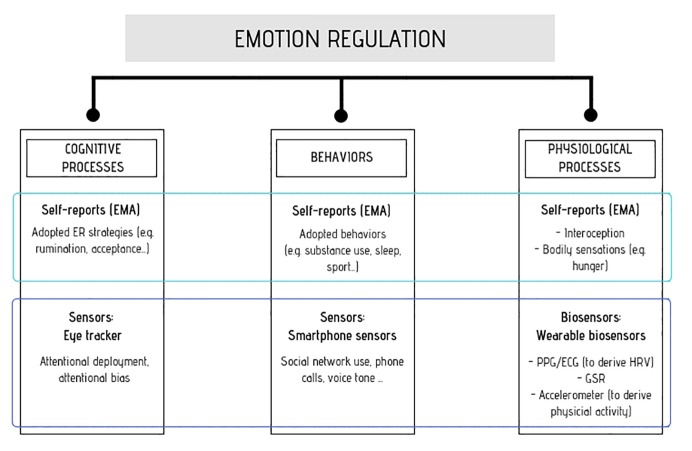
ER is composed of different facets: Cognitive mechanisms, behavioral aspects and physiological processes. The integration of different technologies could grasp all these facets, leading to a more comprehensive understanding of ER. On the one hand, the use of self-reports by means of EMA could assess the three dimensions, i.e., by asking people to report them on a mobile device. On the other hand, the use of embedded-sensors and biosensors could further increase the quantity and objectivity of this information. Cognitive processes, such as attentional deployment or attentional bias, could be investigated using an eye-tracker or other implicit measures like Go/No Go Tasks. Behaviours could be assessed using data gathered from embedded sensors, like number of calls/messages or use of social networks. Finally, the underlying physiological processes could be grasped thanks to wearable biosensors, such as wrist-watches that monitor HRV or GSR parameters. ER, emotion regulation; EMA, ecological momentary assessment; PPG, photoplethysmography; ECG, electrocardiogram; HRV, heart rate variability; GSR, galvanic skin response.

Ideally, an accurate understanding and assessment of ER should not be based only on self-report questionnaires but also on the associated behavioral and physiological mechanisms that could be provided by sensors (Harari et al., 2016) and biosensors (Figure 1; Marzano et al., 2015).

Different studies used mobile phone-embedded sensors data to infer and predict users’ mood (see for example, Ma et al., 2013). Nevertheless, no attempt to integrate self-reports with sensors information has been done in the field of EMA for ER. As a matter of fact, little is known yet about the behavioral consequences or antecedents of adopting certain strategies in daily life and the available limited literature is only based on EMA assessing behaviors through self-reports. Undoubtedly, the behavioral aspect of ER could be further deepened through the use of sensors such as accelerometer, Global Positioning System (GPS), or microphone, which can give us important behavioral information, and that in turn could be very informative of the behavioral antecedents or consequences of the ER strategies (Wampold, 2001). Beyond behaviors, ER-associated cognitive processes could also be grasped by means of the eye-tracker, a tool that allows to measure eyes and gaze movements and, therefore, conscious and/or unconscious attentional deployment. Notably, eye-trackers are nowadays available in portable solutions such as wearable glasses (MacInnes et al., 2018) or smartphone application (Krafka et al., 2016).

Beyond sensors, new breakthrough tools like wristband-like devices or wearable chest straps were created, enabling a noninvasive continuous physiological monitoring outside laboratory settings (Paradiso et al., 2011; Palix et al., 2017). This integration could foster the early detection of dysfunctional patterns. As for the behavioral component, the physiological correlates in daily life are still an understudied aspect of ER. A huge body of studies focused on the physiological markers of ER (Balzarotti et al., 2017), but mainly in laboratory settings, while just few studies investigated these mechanisms in daily life by means of wearable biosensors. In that sense, the combined use of EMA self-reports with daily physiological monitoring already showed its potential in the field of rumination and perseverative negative thinking (Ottaviani et al., 2016), highlighting the association between this strategy and heightened activation of the hypothalamic pituitary adrenal axis (HPAA) and decreased heart rate variability (HRV) during waking, which is considered a robust predictor of maladaptive ER. Although available wearables are still not perfectly accurate for the assessment of physiology in the wild, there are already available options to hurdle this obstacle (for a review of existing wearables, see Peake et al., 2018).

### Virtual Reality

The incorporation of developed VR- and augmented reality (AR)-based tools both for research and practice in different branches of psychology has emerged 20 years ago but steadily increased its preponderance in the last years (Cipresso et al., 2018).

With regard to ER, the assessment by means of VR and AR has an undoubted potentiality although scant research has been conducted yet. VR allows to generate real-life simulated scenarios that may provide a contextualized situation to measure a certain construct through significant environments, though in a controlled way (Riva, 1997, 2009; Riva et al., 2018). This may be the case for ER, given that it would help substantially if a complementary process to the traditional self-report assessment could be carried out by means of the deployment of certain behaviors for which the person involved could spontaneously, and therefore ecologically implement ER strategies that usually are utilized. This may be also of paramount importance to gauge the complexity and diversity that defines the process of ER. Although there are available studies using VR to enhance psychological assessment (Chicchi Giglioli et al., 2015; Cipresso and Riva, 2016; Alcañiz et al., 2018), to the best of our knowledge, just one VR system was specifically developed for ER assessment: The Gameteen System (GT-System). The GT-System is a VR-based serious game to induce negative emotion and train ER strategies in adolescents (Rodriguez et al., 2015). More specifically, negative emotion induction is performed through a “whack a mole” VR-game, which is expressively developed in order to be scarcely accurate and induce frustration. Preliminary results showed that participants’ performance levels were highly correlated with DERS scores, suggesting this game as a possible VR tool to assess ER.

## Intervening in Emotion Regulation Through New Digital Technologies

Interventions targeting ER can be greatly benefited by means of the incorporation of new digital technologies. Among those advantages, a greater dissemination of treatments (Fairburn and Patel, 2017) and the customization of self-help treatments by means of novel statistical procedures are some of the most important (Perna et al., 2018). As for the previous paragraph, we will here provide an overview of current technological applications in the ER field, including mobile applications, internet-based interventions, virtual reality, and biofeedback techniques, showing the advantages of each technology for the research and clinical fields.

### Ecological Momentary Interventions and Health Applications

Ecological Momentary Interventions (EMIs) (Heron and Smyth, 2010) and mental health (mHealth) applications (Naslund et al., 2015) are innovative approaches to provide psychological support through mobile devices in everyday life, without necessarily involving the presence of a real therapist or a face-to-face clinical setting. Among all, the real innovative aspect relies on the possibility of providing personalized and just-in-moment interventions, based on the current needs and affective state of the user.

Many mHealth applications for ER training were developed, mainly focused on cognitive change (Beck, 2017), mindfulness (Plaza et al., 2013), or more generally, on cognitive behavioral therapy (CBT) principles (Rathbone et al., 2017). Nevertheless, most of them do not have a scientific validation or evidence supporting its efficacy (Plaza et al., 2013). Furthermore, the available applications mainly rely on self-reports and do not try to integrate the different dimensions of ER. An innovative recent attempt is represented by Calm Mom, a mobile application that specifically aims at enhancing ER through the integration of data from self-reports and electrodermal activity (Leonard et al., 2018). Due to the continuous EDA monitoring, the application triggers alert when a high level of stress is detected, providing users with a consistent customized ER support (i.e., motivational messages or behavioral strategies).

### Internet-Based Interventions

Under the umbrella of Internet-based Interventions (IBT), a vast array of recent developments are comprised, including computerized and bibliotherapy interventions (Botella et al., 2004; Andersson, 2016). The advantages are many, such as the possibility to reach a great number of people in need that otherwise would not have any kind of access to psychological treatment. Apart from dissemination, internet-based interventions are supposed to increase the cost-effectiveness in comparison with other active treatment (Beecham et al., 2019), although existent literature shows inconclusive results in this regard (Kolovos et al., 2018).

Internet interventions are generally an adaption of classical face-to-face protocols in which emotion regulation plays a relevant role. In this line, different initiatives were carried out taking an already validated protocol and translating the content for an Internet delivery format, many of which entail one or more components to train emotion regulation. The Unified Protocol, one of the first transdiagnostic treatments developed for emotional disorders, which has ER as one of the principal therapeutic targets, constitutes an illustrative example of this (Barlow et al., 2004) and different IBTs have been developed following this model. The first IBT in this line was called Smiling is Fun. A randomized control trial (RCT) with 124 participants showed its effectiveness in reducing depressive symptomatology through the enhancement of ER strategies (Mira et al., 2017). Furthermore, two other RCTs are being conducted. One aims to analyze the effectiveness of a transdiagnostic IBT compared to treatment as usual (González-Robles et al., 2015). The other one studies the differential efficacy of the same transdiagnostic IBT but compared to a transdiagnostic IBT with additional components of positive affect enhancement (Díaz-García et al., 2017).

### Virtual Reality and Serious Games

Despite the potentiality described for the assessment, MR have initially emerged in the psychological realm as a powerful intervention tool for facilitating exposure therapy for specific phobias (Rothbaum et al., 1995; Botella et al., 1998; Riva et al., 1999). From the 90s on, a significant amount of research has yielded evidence on the efficacy of MR, particularly VR, for several clinical conditions (Opriş et al., 2012; Turner and Casey, 2014). With the emergence of low-cost devices as well as massive commercial products, VR has become a more feasible tool to be implemented in clinical contexts (Lindner et al., 2017).

Overall, VR can be of paramount importance for the intervention of ER. Illustratively, VR can be an effective tool for emotions’ induction, and therefore, an innovative and more experiential way to train ER strategies in controlled environments. An illustrative example in this direction was developed by Bosse et al. (2012, 2013), who created a virtual scenario that could provide real-time feedbacks of users’ coping skills based on a ER computational model and on the monitoring of behaviors and physiological parameters. Another interesting study targeted ER skills through VR with the aim of preventing adolescents risk behaviors, and although VR did not turn to be more effective that the non-VR condition, adolescents did attend more sessions and incremented their self-efficacy (Hadley et al., 2018). As previously mentioned, the GT-System is another example of a VR-based serious game to assess but also train ER strategies in adolescents through respiration and attention strategy games, which have been shown to significantly reduce frustration levels (Rodriguez et al., 2015).

Furthermore, novel 360° cameras can easily create immersive 360° videos that can be explored from all angles of recording. These videos can be integrated and manipulated by means of software like InstaVR or Google toolkit creator in order to elaborate *ad hoc* scenarios in which to simulate eliciting situations and improve, for example, ER strategies. However, there are also 360° videos in different web platforms that can be downloaded for free. Notably, Li et al. validated a public set of 360° videos for valence and arousal dimensions that can be easily used to experimentally induce emotions (Li et al., 2017a).

Finally, the incorporation of gamified features in the context of treatments can be of tremendous help, not only for the increase of engagement (Looyestyn et al., 2017) but also for the use, both at an experimental and intervention level. A recent systematic review has synthesized the evidence of studies exploring the connection between videogames and ER, showing that there are 23 studies that have explored this in the context of commercial and bespoke games (Villani et al., 2018). Although the review grasps a broad concept of ER, including emotional and mood regulation and even stress responses, it is a first approximation to better understand how such an ecological stimulus for young, adolescents, and event adults may be incorporated in the current perspective of ER assessment and intervention.

### Biofeedback

Biofeedback constitutes an effective and noninvasive procedure, whose basic operating principle is the conscious registration of normally unconscious body procedures (e.g., brain activity, electrocardiogram, electromyography, or skin conductance) (Gaume et al., 2016) that are represented by a visual, haptic, or audio signal. As aforementioned, there is a large body of evidence showing the strong relation of ER with physiological processes such as HRV (Appelhans and Luecken, 2006; Balzarotti et al., 2017). Precisely, HRV biofeedback has shown to be effective for stress and anxiety (Goessl et al., 2017), conditions that have shown to be greatly explained by emotion regulation (Barlow et al., 2004). Besides, the neural activity, in particular, the activity of the amygdala, which constitutes a key area for emotion activity and regulation, has shown to be successfully regulated through neurofeedback procedures (Johnston et al., 2010; Zotev et al., 2013; Zich et al., 2018).

Finally, the integration of biofeedback with VR constitutes a very powerful research line. It would permit to provide users with engaging interfaces of the physiological targeted stimuli, which could in turn positively impact on the therapeutic outcomes. As an example, Lorenzetti and colleagues (Lorenzetti et al., 2018) implemented a real-time functional magnetic resonance imaging neurofeedback protocol to enhance emotional states in healthy subjects.

## Future Perspectives in the Incorporation of New Technologies for Emotion Regulation

All the described developments show that new digital technologies not only constitute a potentiality but also an already current way to improve our knowledge of ER. Specifically, the following aspects are the most relevant to take into consideration for the further developments within the field.

### Ecological Validity

In ER research field, many smartphone-based EMAs have been adopted in order to ecologically investigate ER in daily life, showing the potentialities of this approach to grasp emotion dynamics in real-life settings. However, few studies integrated self-reports with data gathered from embedded-sensors or wearable biosensors, which could instead bring new insights into ER daily processes in terms of determinants and consequences as well as contextual factors affecting ER. Notably, no EMA for ER assessment has been developed so far with the aim of being applied to clinical practice, where retrospective self-reports are still the most used method. We suggest that EMA could increase assessment accuracy by considering ER as a situated process, in which momentary and contextual factors play a key role. This would, in turn, help clinicians identify the strategies to be addressed by the therapy as well as the potential triggers of maladaptive strategies. Similarly, VR could constitute a powerful tool to develop realistic scenarios in which to elicit emotions and explore/assess ER deployment as well as train ER strategies. Nevertheless, this field is still completely understudied.

### Individual Differences

In line with the first point, the potentiality of utilizing embedded sensors and wearable biosensors along with complex machine learning techniques is still undeveloped in the specific case of EMI for ER. Its progress could lead to the development of aware systems able to more accurately explore and predict users’ emotion and affect regulation and provide support in specific moment of the day (Kuppens, 2015). Furthermore, VR could also be a powerful tool to increase intervention personalization. The possibility of manipulating VR scenarios could indeed represent a new way of intervening in ER, where it would be possible to adapt environments to the needs and characteristics of each individual.

### Overcoming a Schematic Study of Single Emotion Regulation Strategies

Instead, the previously cited possibility of gathering large amount of data may allow grasping the complex interplay of the different strategies. In this way, the commonly used classification of adaptive or maladaptive strategies may be left aside in order to incorporate a context-based perspective. An integration of implicit, automatic, and interpersonal ER processes could also be achieved through an articulated study of cognitive, behavioral, experiential, and psychophysiological dimensions by means of the incorporation of all the described technologies.

### Integrated Knowledge of ER

An integrated understanding of ER (i.e., implicit, automatic, and interpersonal dimensions) could be achieved through an articulated study of cognitive, behavioral, experiential, and psychophysiological dimensions by means of the incorporation of all the described technologies. For instance, a wide range of technologies such as VR-based avatars could embrace an embodied cognition perspective (Bailey et al., 2016), which is also an essential aspect within the emotion regulation field (Koole and Veenstra, 2015).

**Figure 2:**
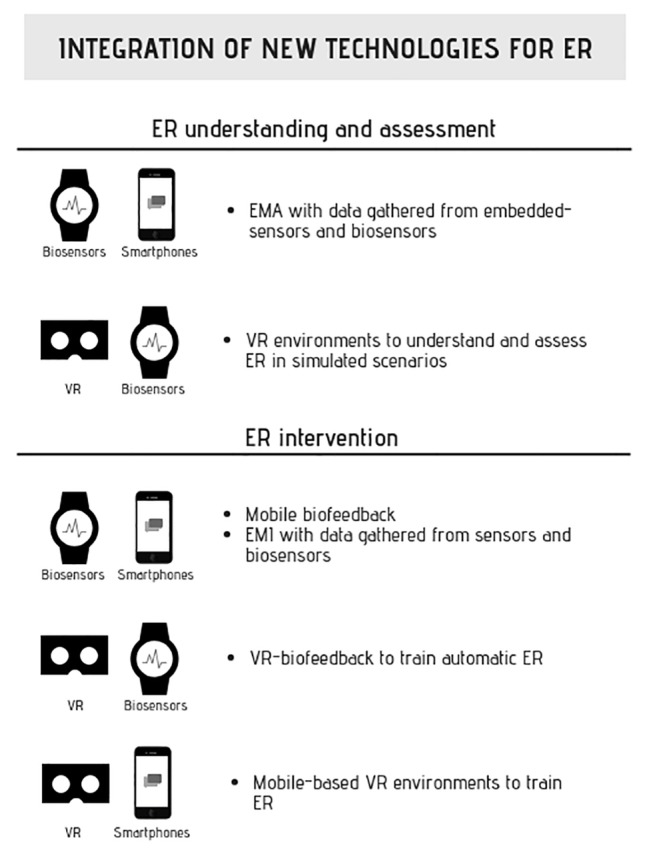
Examples of the complementary and integrative role f new technologies for the understanding assessment and intervention of ER. ER, emotion regulation; EMA, ecological momentary assessment; VR, virtual reality; EMI, ecological momentary intervention.

For the aforementioned reasons, the pursuit of integrated prototypes of technologies could lead to a successful understanding, assessment, and training of ER (Figure 2). All these advancements should be conducted in a multidisciplinary way, i.e., in active collaboration with the latest Human Computer Interaction and Biomedical Engineering findings. Ongoing examples of integrated technologies are already occurring, like the development of an interpersonal VR-based biofeedback called “DYNECOM” (Salminen et al., 2018). DYNECOM is an immersive VR system for the practice of empathy-evoking compassion meditation by dyads (Salminen et al., 2018). Within the virtual environment, real-time EEG and breath rate visual feedbacks are provided, as well as a visual representation of the signals synchronization between participants. According to preliminary results, this innovative couple biofeedback is able to increase the perceived affective interdependence between the dyad, suggesting the potentiality of this device to improve implicit ER. Other examples are VR-based intrapersonal biofeedback (Gaggioli et al., 2014), a mobile biofeedback with serious games (Dillon et al., 2016), gamified virtual reality (Miloff et al., 2016), or fMRI neurofeedback in virtual environments (Lorenzetti et al., 2018). If this integration is further developed, a powerful path for the upcoming years can be expected, resulting in the enhancement of the field of emotion regulation.

## Author Contributions

JF-Á and DC developed the idea for this perspective and equally contributed to the conceptualization and writing—original and subsequent drafts until last version. PC, AGP, CB, and GR contributed to writing—review, conceptualization, editing, and supervision.

### Conflict of Interest Statement

The authors declare that the research was conducted in the absence of any commercial or financial relationships that could be construed as a potential conflict of interest.
